# NADPH-Cytochrome P450 Reductase: Molecular Cloning and Functional Characterization of Two Paralogs from *Withania somnifera* (L.) Dunal

**DOI:** 10.1371/journal.pone.0057068

**Published:** 2013-02-21

**Authors:** Satiander Rana, Surrinder K. Lattoo, Niha Dhar, Sumeer Razdan, Wajid Waheed Bhat, Rekha S. Dhar, Ram Vishwakarma

**Affiliations:** 1 Plant Biotechnology, Indian Institute of Integrative Medicine (CSIR), Canal Road, Jammu Tawi, India; 2 Medicinal Chemistry, Indian Institute of Integrative Medicine (CSIR), Canal Road, Jammu Tawi, India; Cankiri Karatekin University, Turkey

## Abstract

*Withania somnifera* (L.) Dunal, a highly reputed medicinal plant, synthesizes a large array of steroidal lactone triterpenoids called withanolides. Although its chemical profile and pharmacological activities have been studied extensively during the last two decades, limited attempts have been made to decipher the biosynthetic route and identification of key regulatory genes involved in withanolide biosynthesis. Cytochrome P450 reductase is the most imperative redox partner of multiple P450s involved in primary and secondary metabolite biosynthesis. We describe here the cloning and characterization of two paralogs of cytochrome P450 reductase from *W. somnifera*. The full length paralogs of *WsCPR1* and *WsCPR2* have open reading frames of 2058 and 2142 bp encoding 685 and 713 amino acid residues, respectively. Phylogenetic analysis demonstrated that grouping of dual CPRs was in accordance with class I and class II of eudicotyledon CPRs. The corresponding coding sequences were expressed in *Escherichia coli* as glutathione-*S*-transferase fusion proteins, purified and characterized. Recombinant proteins of both the paralogs were purified with their intact membrane anchor regions and it is hitherto unreported for other CPRs which have been purified from microsomal fraction. Southern blot analysis suggested that two divergent isoforms of CPR exist independently in *Withania* genome. Quantitative real-time PCR analysis indicated that both genes were widely expressed in leaves, stalks, roots, flowers and berries with higher expression level of *WsCPR2* in comparison to *WsCPR1*. Similar to CPRs of other plant species, *WsCPR1* was un-inducible while *WsCPR2* transcript level increased in a time-dependent manner after elicitor treatments. High performance liquid chromatography of withanolides extracted from elicitor-treated samples showed a significant increase in two of the key withanolides, withanolide A and withaferin A, possibly indicating the role of *WsCPR2* in withanolide biosynthesis. Present investigation so far is the only report of characterization of CPR paralogs from *W. somnifera*.

## Introduction

*Withania somnifera* (L.) Dunal commonly known as ashwagandha or winter cherry, belongs to family Solanaceae. Its versatile reproductive strategy of mixed mating and robust chemotypical variability enables it to thrive under marginal and xeric habitats with a wide geographic distribution [1], [2]. It is a multipurpose medicinal plant, synthesizing large array of bio-active secondary metabolites. In recent years there has been a remarkable surge in the pharmacological studies of this plant as it has been shown to possess wide spectrum of therapeutic properties including antitumor, immuno-modulatory, chemo-protective, cardio-protective and neuroprotective [3], [4], [5]. Most of the biological activities are attributed to naturally occurring C-28 steroidal lactone triterpenoids built on an intact or rearranged ergostane framework, in which C-22 and C-28 are oxidised to form a 6-membered lactone ring. These steroidal triterpenoids known as withanolides have a structural resemblance with active constituents of *Panax ginseng* called gingosides. *W. somnifera* also known as Indian ginseng is often compared with Korean ginseng (*P. ginseng*) for its rejuvenating properties. Withanolides are synthesized and accumulated in leaves and roots of *W. somnifera*. Chemoprofiling of *Withania* presents several alkaloids, sitoendosides, and more than 40 withanolides [6], [7]. Substantial pharmacological activities have been accredited to two main withanolides, withaferin A (WS-3) and withanolide D (WS-D). These compounds have been reported to inhibit angiogenesis, Notch-1, NFκB in cancer cells and induce apoptosis in breast cancer cells [8], [9], [10].

**Figure 1:**
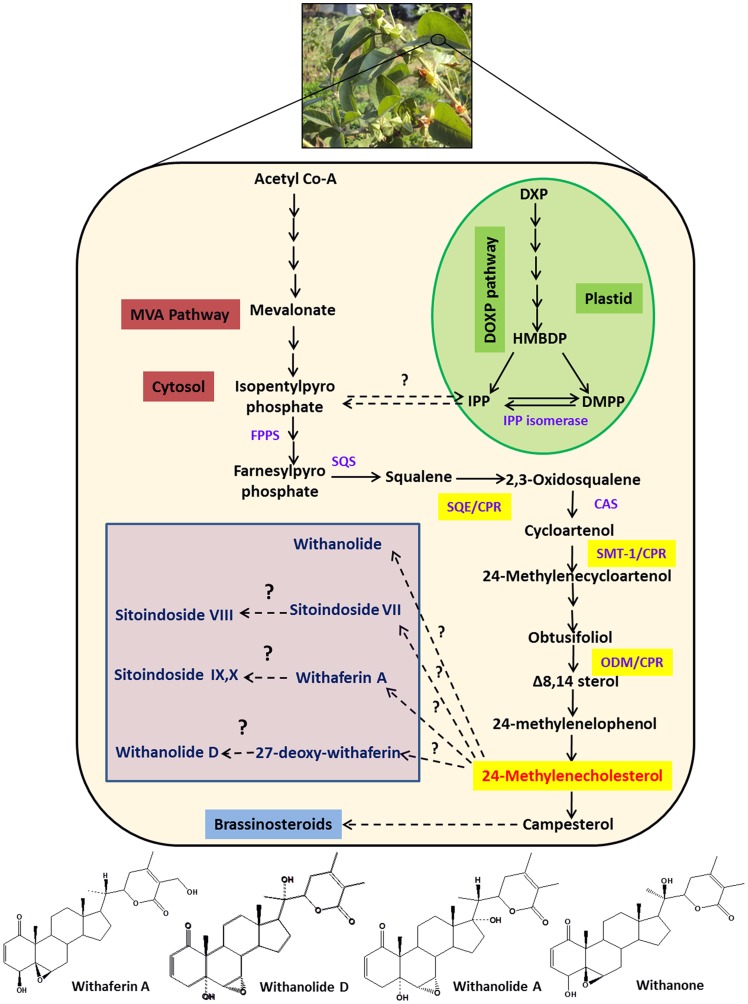
An overview of putative withanolide biosynthesic pathway. DXP: 1-deoxy-D-xylulose 5-phosphate, HMBDP: 1-hydroxy-2-methyl-2-(E)-butenyl 4-diphosphate, IPP:Isopentylpyrophosphate, DMPP: Dimethylalyl diphosphate, IPP isomerase: Isopentylpyrophosphate isomerase, FPPS: farnesyldiphosphate synthase, SQS: Squalene synthase, SQE/CPR: Squalene epoxidase/cytochrome P450 reductase, CAS: Cycloartinol synthase, SMT-1: Sterol methyl transferase/cytochrome P450 reductase, ODM/CPR: Obtusifoliol-14-demethylase/cytochrome P450 reductase. First three highlighted (yellow) steps indicating involvement of P450 monooxygenases and CPR. Single dark arrows represent one step, two or more dark arrows represent multiple steps and dashed arrow represents unknown steps. Below the pathway: chemical structure of important withanolides.

Principally, withanolides (C-30) are synthesized *via* both mevalonate (MVA) and non-mevalonate (DOXP) pathways. The head-to-tail condensation of isopentenyl pyrophosphate (IPP) leads to formation of farnesyl diphosphate (FPP) which is the main precursor for triterpenoids [11], [12]. A key intermediate compound, 24-methylenecholesterol is an immediate precursor for biosynthesis of different withanolides (Figure 1). While there is considerable literature pertaining to the fascinating chemistry and biological studies of many withanolides from *W. somnifera*, but sparse information exists regarding their biosynthesis.

From biochemical, molecular and pathway engineering perspectives, it is imperative to characterize key pathway genes and to understand their regulatory role in the biosynthesis of bioactive metabolites. Cytochrome P450 enzymes belong to one of the largest and most functionally diverse protein super-families which is pivotal in various metabolic molecular circuitries. P450s are heme thiolate-proteins, catalyse extremely diverse variety of reactions like hydroxylations, dealkylations, sulphoxidations, epoxidations, reductive dehalogenations, peroxidations and various types of isomerization for the synthesis of a suite of primary and secondary metabolites essential for plant growth and development [13], [14]. P450 monooxygenases constitute a substrate specific class of enzymes which are highly regio and stereo-specific. By gene annotation approximate 1% of the total genes in the plant’s genome are found to be cytochrome P450s. *Arabidopsis* genome contains 244 genes and 28 pseudo-gene representing cytochrome P450s [15]. P450s are located in the endoplasmic reticulum (ER) and their catalytic activities strictly rely on supply of electrons from NADPH:cytochrome P450 reductase (CPR: diflavoenzyme).

CPRs (EC 1.6.2.4) are membrane bound proteins localized to ER, contain an N-terminal positioned FMN binding domain linked to NADPH binding domain *via* FAD domain. CPR shuttles electrons derived from NADPH through FAD and FMN domains into the heme iron-centre of the various P450 enzymes. Genes encoding CPR from many species of animals, insects and yeast have been isolated and among all only single form is known to interact with various P450s [16]. However, in vascular plants number of CPR paralogs vary from one to three depending on the species e.g. *Nothapodytes foetida* and Hybrid poplar (*Populus trichocarpa* × *Populus deltoides*) [17], [18] contain three paralogs while *Helianthus tuberosus*, *Petroselinum crispum*, *Arabidopsis thaliana, Centaurium erythrae* and *Gossipium hirusitum* have two CPR homologs. [19], [20], [21], [22], [23]. Similarly, single form of CPR has been characterised from some plants like *Coleus blumei*, *Papaver somniferum*, *Taxus cuspidate*, *Vigna* radiate [24], [25], [26], [27]. Based on the N- terminal anchoring sequences, Ro *et al.* [18] have classified CPRs into two classes: Class I and class II. CPR1 belonging to class I is found to express constitutively while in class II, CPR2 is transcribed under stress or elicited by wounding or elicitors. Presence of multiple CPRs in plants may reflect the diversity of P450s [28], [29] and seem primarily to confront the high demand of electron supply during biotic and abiotic stress or differential expression at various stages of plant development [18], [21].

Biochemical and molecular studies have been recently initiated to elucidate biosynthetic pathway for various withanolides in *W. somnifera* [30]. As a part of on-going endeavour to investigate various pathway genes in withanolide biosynthesis, we in present study report cloning and characterization of two paralogs of CPR (NCBI GenBank *Acc No. WsCPR1*: HM036710 and *WsCPR2*: GU808569). The investigations also include comparative tissue-specific expression analysis of *WsCPR1* and *WsCPR2* and changes in three key withanolides namely withanolide A (WS-1), withanone (WS-2) and withaferin A (WS-3) in response to elicitor stimuli methyl jasmonate (MeJA) and salicylic acid (SA). There are numerous studies related to heterologous cDNA-expression to identify different physiological substrates for proteins in the P450 superfamily. The heterologous expression of P450s is often constrained because of inadequate interface of endogenous cytochrome P450 oxido-reductase partners resulting in no or low activity [31]. We tend to understand the regulatory role by co-expression of cloned CPRs with different P450 monooxygenases which have been demonstrated to result in increased utilization of substrate and found to enhance the production of secondary metabolites. Different paralogs of CPR have been implicated to possess diverse physiological roles particularly in secondary metabolism, plant adaptation and defence mechanism by maintaining electron supply to different P450 monooxygenases [32].

## Materials and Methods

### Plant Material

A WS-3 rich genetic stock of *W. somnifera* designated as WS-Y-08 (described elsewhere [30]) grown at IIIM experimental farm (Indian Institute of Integrative Medicine, CSIR, Jammu, India, 32°44′N longitude, 74°55′E latitude; 305 m in altitude) was used as a source material. *In vitro* raised plants through forced axillary bud culture of accession WS-Y-08 were used for MeJA and SA treatments. Samples were collected, frozen immediately in liquid nitrogen, and stored at −80°C.

### RNA Isolation and cDNA Synthesis

Total RNA was extracted using Trizol reagent (Sigma, St. Louis, USA) as per manufacturer’s protocol. Concentration of isolated RNAs was estimated by measuring the absorbance at 260 nm in a spectrophotometer (AstraAuriga, Cambridge, UK). Further, quality of RNA was assessed by determining the ratio of absorbance at 260 and 280 nm (*A*_260/280_) and formaldehyde-denatured agarose gel electrophoresis. Total RNA (5 µg) was incubated with RNase free DNase (Fermentas, Burlington, Canada) at 37°C for 30 min. First strand cDNA synthesized using the RevertAid cDNA synthesis kit (Fermentas, Burlington, Canada) in a total volume of 20 µl containing 3 µg total RNA, 10 mM dNTPs, 10 µM oligo (dT) primer, 1 µl M-MuLV reverse transcriptase (200 U/µl) and 1× first strand buffer (250 mM Tris-HCl, pH 8.3, 250 mM KCl, 20 mM MgCl_2_, 50 mM DTT). The reaction was incubated for 60 min at 42°C followed by 5 min at 70°C to inactivate the reverse transcriptase.

### Cloning of *WsCPR1* and *WsCPR2*

Degenerate primers (Table 1) based on the conserved regions of CPRs were designed by multiple sequence alignment of different CPR sequences retrieved from the GenBank database at National Center for Biotechnology Information (NCBI). Optimization of reverse transcriptase-polymerase chain reaction (RT-PCR) conditions allowed amplification of cDNA fragments corresponding to *WsCPR1* and *WsCPR2* under following cycling conditions: one cycle of 94°C for 3 min, 35 cycles of 94°C for 30 s, 55°C for 1 min and 72°C for 2 min followed by a final extension of 72°C for 10 min in a thermal cycler (Bio-Rad Laboratories, Hercules, CA, USA). Amplicons examined by agarose gel electrophoresis were cloned into pTZ57R/T vector (Fermentas, Burlington, Canada), and then transformed into *E. coli* DH5α host strain. Cloned amplicons were sequenced using an automated DNA sequencer (ABI Prism 3130XL; Applied Biosystems, Foster City, CA, USA). The nucleotide sequences obtained were analysed using the similarity search BLAST program and subsequently used for designing gene specific primers (GSPs).

**Table 1:** List of primers used in the study.

Primers	Sequence (5′−3′)		Direction
**Degenerate**			
PLCPR -1F	GAGCCIACIGATAATGCTGCN(A/C/T/G)A(C)G		Forward
PLCPR-1R	TTGCY(C/T)GAW(A/T)GGR(A/G)AAM(C/A)CAGCCA		Reverse
PLCPR-2F	GTCATGGS(G/C)K(T/G)GAATTCCW(A/T)TCAG		Forward
PLCPR-2R	GGACCY(T/C)GAR(G/A)AAR(G/A)GCWAC		Reverse
**5′ and 3′ RACE**			
5′ Adapter*		GCUGAUGGCGAUGAAUGAACACUGCGUUUGCUGGCUUUGAUGAAA	
5′ RACE-OUT*		GCTGATGGCGATGAATGAACACTG	Forward
5′ RACE-IN*		CGCGGATCCGAACACTGCGTTTGCTGGCTTTGATG	Forward
5′ CPR1-OUT		CATCATCGGCAGCATAATCATCCAT	Forward
5′ CPR1-IN		CAACTGCCTTCTCATACCTTGCT	Forward
5′ CPR2-OUT		GCCATCCTAGGAGATGATGAGATA	Reverse
5′ CPR2-IN		ACTGAAGGCTTGGCTGATGGAAAT	Reverse
3′ Adapter*		GCGAGCACAGAATTAATACGACTCACTATAGGT_12_V(G/A/C)N(A/C/T/G)	
3′ RACE-OUT*		GCGAGCACAGAATTAATACGACT	Reverse
3′ RACE- IN*		CGCGGATCCGAATTAATACGACTCACTATAGG	Reverse
3′CPR1-OUT		GATCAGATACTTAGAGACGAGGATG	Forward
3′ CPR1-IN		TACGGCTGCAATTCCTGAATATCG	Forward
3′ CPR2-OUT		AGCTAGTTGTTGCTTTCTCACGTG	Forward
3′ CPR2-IN		CTAACAAACAATACGTGCAGCATA	Forward
Full length			
FullCPR1F		ATGGAGTTGAGTTCGGAGTTGGTGAG	Forward
FullCPR1R		TCACCACACATCCCTGAGATATCTTCC	Reverse
FullCPR2F		ATGGACTCTACATCAGAAAAACTTTCTCC	Forward
FullCPR2R		TCACCACACATCACGCAGATATCT	Reverse
**Expression**			
CPR1*BamHI*F		CGGGATCCATGGAGTTGAGTTCGGAGTTGGTGAG	Forward
CPR1*XhoI*R		CCGCTCGAGATCACCACACATCCCTGAGATATCTTCC	Reverse
CPR2*BamHI*F		CGGGATCCATGGACTCTACATCAGAAAAACTTTCTCC	Forward
CPR2 *NotI*R		ATTTGCGGCCGCTCACCACACATCACGCAGATATCT	Reverse
**Real-Time analysis**			
RTCPR1F		AGCAAGGTATGAGAAGGCAGTTGT	Forward
RTCPR1R		CAGCATTATCAGTTGGCTCACCAT	Reverse
RTCPR2F		AGTGTGGCCTAAATTGGATAAGTTGC	Forward
RTCPR2R		ACGATAACATGTCCATTTGCATGAC	Reverse
RTActinF		GAGAGTTTTGATGTCCCTGCCATG	Forward
RTActinR		CAACGTCGCATTTCATGATGGAGT	Reverse

* Primers marked with star were provided with the kits.

### 5′ and 3′ RACE

To validate prediction of target genes, 5′ & 3′ rapid amplification of cDNA ends (RACE) were performed using first choice RLM-RACE kit according to the product manual (Ambion, Austin, TX, USA). cDNAs obtained were subjected to nested PCR using GSPs as listed in the Table 1. Two rounds of PCR were performed, first round with 5′ RACE-OUT corresponding to the 5′ RACE adapter sequence and 5′ CPR1-OUT and 5′ CPR2-OUT as specific outer primers, followed by a second round of PCR with 5′ RACE-IN and 5′ CPR inner GSPs. In both rounds, PCR reactions of 50 µl containing 1.0 µl cDNA as template (except for second round where amplified products of 1st round were used as template), 2 µl of 10 µM 5′ CPR1-OUT and 5′ CPR2-OUT, 2 µl of 5′ RACE outer, 45.0 µl master Mix (34.5 µl PCR-grade water, 10 mM Tris HCl; pH 9.0, 50 mM KCl, 2.5 mM MgCl_2_, 200 µM dNTPs, 2.5 U Taq DNA polymerase) were subjected to following cycling conditions: One cycle of 94°C for 3 min and 35 cycles of 94°C for 30 s, 60°C for 30 s, 72°C for 2 min with a final step at 72°C of 10 min. Similarly for 3′ RACE, first strand cDNA was synthesized from total RNA using supplied 3′ RACE adapter primer. cDNA was subjected to nested PCR using outer and inner primers specific to 3′ RACE adapter along with 3′ GSPs with same reaction volume and cycling conditions as described for 5′ RACE. All PCR products were gel purified, cloned and sequenced.

### Full-length Cloning of *WsCPR1* and *WsCPR2*

By comparing and aligning the sequences of the core fragments, 5′ RACE and 3′ RACE products, the full-length cDNAs of *WsCPR1* and *WsCPR2* were generated and subsequently amplified with primers FullCPR1F, FullCPR1R and FullCPR2F, FullCPR2F (Table 1). A high fidelity proof-reading DNA polymerase (New England Biolabs, Herts, UK) was employed for amplification under PCR conditions; One cycle of 94°C for 3 min, 35 cycles of 94°C for 30 s, 60°C for 30 s, 72°C for 2 min. The final extension was at 72°C for 10 min. PCR products were analysed on 1.2% agarose gels and visualized under UV light. The resulted amplified products were ligated in pJET vector and sub-cloned into *E.coli* DH5α.

### Sequence Analysis

BLAST (http://www.ncbi.nlm.nih.gov) was used to find similarity of amplified CPRs in Genbank database. Translate tool (http://www.expasy.ch/tools/dna.html) was used to predict the open reading frame (ORF). The properties of deduced amino acid sequences of *WsCPR1* and *WsCPR2* were estimated by using ProtParam (http://www.expasy.ch/tools/protparam.html), SPLIT v.4.0 (http://split.pmfst.hr/split/4/) and TMHMM (http://www.cbs.dtu.dk/services/) programs. Predictions of N-glycosylation and chloroplast targeting sites in both polypeptides were computed using NetNGlyc 1.0 Server (http://www.cbs.dtu.dk/services/NetNGlyc/) and ChloroP (http://www.cbs.dtu.dk/services/ChloroP). ClustalX was used for multiple sequence alignment [33]. Protein secondary structures were determined by SOPMA program [34]. Structurally and functionally important regions were identified in deduced protein sequence by Conseq services. To assess the evolutionary relationships between the *WsCPRs* and CPR homologs from different plants species, sequences were retrieved using BLASTp searches using *WsCPR1* and *WsCPR2* as query. Further, sequences were aligned using the ClustalW (http://www.ebi.ac.uk) and a phylogenetic tree was constructed by Neighbour-joining method using MEGA 5 software. Bootstrap analysis with 100 replicates was also conducted in order to obtain confidence levels for the branches.

### Prediction of Three-dimensional Structures of *WsCPR1* and *WsCPR2*

Three-dimensional structures of *WsCPR1* and *WsCPR2* were predicted using Phyre^2^ [35] with the crystal structure of *Rattus norvegicus* (PDB ID: 1J9Z) as a template. Ligand binding sites were predicted using 3DLigandSite [36]. Conserved amino acids at the protein surface were determined using ConSurf (http://consurf.tau.ac.il/overview.html).

### Heterologous Expression of Recombinant Proteins

Full length coding sequences of *WsCPR* genes were modified by adding restriction sites (Table1). Immediately upstream to start codons, *BamHI* in both genes and downstream to stop codons, *XhoI* (*WsCPR1*) and *NotI* (*WsCPR2*) restriction sites were introduced using engineered sense and anti-sense primers (Table 1). Using directional cloning, ORFs of candidate P450 reductases were excised from pJET and transferred to pre-digested, purified pGEX4T-2. The *E.coli* BL21 (DE3) cells were transformed with pGEX-*WsCPR1* and pGEX-*WsCPR2* expression cassettes. A single colony of each recombinant culture was inoculated separately into 100 ml of Luria–Bertani (LB) broth containing 100 µg/ml of ampicillin and incubated overnight at 37°C. 1% culture was transferred into 100 ml of LB media containing the corresponding antibiotic and incubated at 37°C, until optical density (*A*_600 nm_) reached 0.4–0.5. Protein expression was induced by adding Iso-propyl β-D-1-thiogalactopyranoside (IPTG; Fermentas, Berligton, Canada) into the cultures at the concentration of 0.2 mM to 1 mM. The cultures were constantly incubated at 25°C for 8–12 h. The induced bacterial cells were harvested at an interval of 2 h by centrifugation and resuspended in 6x sodium dodecyl sulphate–polyacrylamide gel electrophoresis sample buffer (SDS-PAGE; 0.375 M Tris pH 6.8, 12% SDS, 60% glycerol, 0.6 M DTT, 0.06% bromophenol blue). The expression of target proteins was analysed on 10% SDS-PAGE.

### Purification of *WsCPR1* and *WsCPR2*

Protein expression was induced by addition of 0.8 mM IPTG when the optical density at 600 nm reached 0.4. Cells were grown for further 8–12 h at 25°C and then harvested by centrifugation (6000 *g* at 4°C for 10 min; Eppendorf, Hamburg, Germany). Pelleted cells were resuspended in the 1× PBS (140 mM NaCl, 2.7 mM KCl, 10 mM Na_2_HPO_4_, 10 mM KH_2_PO_4_, pH 7.3) and lysed by adding 100 mM DTT followed by lysozyme (100 µg/ml) for 45 min at 4°C. The lysates were sonicated four times for 30 s each time (amplitude 1, 20% duty cycle) using a sonicator (Sartorius, Gottingen, Germany). To the lysates, 10% triton X*-*100 was added and incubated for 30 min at 4°C. Further, soluble and insoluble fractions were separated by centrifugation (14, 5000 *g* at 4°C for 15 min). The supernatant was incubated overnight with glutathione-sepharose beads (1 ml L^−1^ of culture) (GE Healthcare, Little Chalfont, UK) at 20°C. The beads were washed five times with 10 bead volumes of 1× PBS. To remove the glutathione *S*-transferase (GST) moiety, thrombin (4 U/ml of beads) was added to the beads, and cleavage was allowed to proceed for 10–12 h at 24°C. The beads were pelleted (600 *g* at 4°C for 5 min), supernatant containing proteins were incubated overnight further with benzymedene beads to remove the thrombin. The purified protein samples were denatured and analysed on 10% SDS-PAGE and their concentration was directly measured on spectrophotometer.

### Southern Blotting

The genomic DNA was isolated from mature leaf-tissue using Qiagen DNeasy plant mini kit (Qiagen, Hilden, Germany) following manual provided and quantified at *A*_260/280 nm_. Aliquots of DNA (30 µg) were digested for 16 h at 37°C with *BamHI*, *NotI* and *BglII* for *WsCPR1* and *NotI*, *PvuI* and *EcoRI* for *WsCPR2* respectively. Digested products were electrophoresed on 0.8% agarose gel and transferred onto positively charged nylon membrane (Roche, Manheim, Germany) by capillary blotting [37]. The probes for southern hybridization were synthesized by PCR using full length primers that amplified the coding sequence of *WsCPR1* and *WsCPR2* respectively. The probes were labelled with digoxigenin (DIG)-dUTP using the DIG-DNA synthesis Kit (Roche, Manheim, Germany) according to the manufacturer’s instructions. The blot was hybridised, blocked and incubated with antibodies. Further, it was washed and signal generation and detection were performed according to the manufacture’s manual.

### Tissue-specific Expression Analysis

To study tissue-specific expression, total RNA was extracted from leaves, stalks, roots, flowers and berries respectively. Total RNA (5 µg) was incubated with RNase free DNase (Fermentas, Burlington, Canada) at 37°C for 30 min. cDNA was synthesized from 3 µg of RNA using RevertAid cDNA synthesis kit according to manufacturer’s instruction. SYBR green PCR amplification was performed using the StepOne Real-time PCR System (Applied Biosystems, Foster City, CA, USA). Briefly, for the standard 20 µl of reaction, included 0.2 µL cDNA template, 200 nM each of the primers, and 10 µL SYBRPremix Ex **(**Takara, Otsu, Japan**)** under following cycling conditions: one cycle of 94°C for 1 min, 40 cycle of 94°C for 10 s, 60°C for 20 s and 72°C for 25 s. Two primers, Actin F and Actin R were used to amplify housekeeping actin gene as control. All samples were analysed in triplicate and the specificity of each primer pair was validated by a dissociation curve (a single peak was observed for each primer pair). Data obtained were subjected to analysis using StepOne software v 2.0.

### Elicitor Treatment

For elicitor treatment the micro-propagated plantlets were pre-cultured in Hoagland’s suspension medium for 2 wk. The plantlets were treated with MeJA (0.1 mM) and SA (0.1 mM) dissolved in dimethylsulphoxide (DMSO) and untreated kept as control with same amount of DMSO. The tissue from each treated sample was harvested after 6, 12, 24 and 48 h for RNA isolation and withanolides extraction. cDNA was obtained from each treated sample including control, using the same RNA isolation and cDNA synthesis protocols as described above. Effect of elicitor treatment on expression profile was studied using semi-quantitative PCR. The PCR cycles for each gene were optimized to their exponential stage. Actin was used as an internal control. The reaction mixture for each sample contained 10 mM Tris HCl pH 9.0, 50 mM KCl, 2.5 mM MgCl_2_, 200 µM dNTP, 1 µM RT primers (Table 1), 0.5 µl of cDNA template, and 0.5 U of *Taq* DNA polymerase (Fermentas, Burlington, Canada). The PCR conditions were as follows: one cycle 94°C for 1 min, 27 cycles of 94°C for 30 s, 60°C for 20 s and 72°C for 25 s. The samples were analysed on 1.5% agarose gel and densitometric quantitation of relative band intensities by normalizing with actin was performed using QuantityOne v 3.0 software (Bio-Rad, Laboratory, Philadelphia, USA).

### Extraction and Quantification of Withanolides Using HPLC

Withanolides were extracted and quantified as described by Dhar *et al*. [38]. Concisely, samples of *W. somnifera* were powdered and extracted with ethanol-water (50∶50; *v/v*) with magnetic stirring at room temperature (25±2°C). Extracts were filtered and the solvent was removed under vacuum. The extracts (20 mg/ml) obtained from each sample of the plant material were prepared in HPLC-grade methanol-water (50∶50; *v/v*) for quantitative analysis. 1.2 mg per 2 ml of the standards of withanolide A (WS-1), withanone (WS-2) and withaferin A (WS-3) were prepared in HPLC-grade methanol. HPLC analysis was performed with Shimadzu HPLC system (Shimadzu, Tokyo, Japan) equipped with 515 quaternary gradient pump, 717 Rheodyne injector, 2996 PDA detector and CLASS-VP software v 6.14. All samples were filtered through 0.45 µM filters (Millipore, Bedford, USA). Extracts of *W. somnifera* samples were separated on a RP-18e (4.6×100 mm, 5 µm) (Merck, Bangalore, India) column. The mobile phase consisted of methanol-water (60∶40; *v/v*) delivered at a flow rate of 0.5 ml/min. The samples were analyzed at 30°C to provide efficiency to the peaks. The UV chromatograms were recorded at 237 nm.

### NADPH-reductase Assays

Activities of purified NADPH- reductases were determined by the reduction of cytochrome *c* (20 µM) in presence of NADPH (100 µM). All assays and incubations were carried out in 300 mM potassium phosphate buffer, containing 0.1 mM EDTA, pH 7.8 at 25°C. The rate of reduction was monitored by increase of absorbance at 550 nm [39]. For calculating the reduction rate of cytochrome *c*, a specific molar absorption coefficient (21.1 mM^−1^ cm^−1^) of equine heart cytochrome *c* was used. Further, for measurement of kinetic parameters, NADPH (100 µM) with varying concentrations of cytochrome *c* (10–250 µM) were used in the reaction mixture. The kinetic constants K_m_ and V_max_, were calculated with non-linear regression analysis using GraphPad Prism 5 software.

## Results and Discussion

### Cloning of Full Length cDNAs Encoding *WsCPR1* and *WsCPR2*

Plant P450s catalyse a diverse array of reactions during secondary metabolite synthesis which require involvement of their redox partner, NADPH:cytochrome P450 reductase [40]. In this investigation, two CPR paralogs from *W. somnifera, WsCPR1* and *WsCPR2* were isolated and expressed in *E. coli* BL21 (DE3) to study their catalytic properties. Gene expression patterns were also studied in relation to elicitors (MeJA & SA) and corroborated with chemo-profiles.

Full length cDNAs of *WsCPR1* and *WsCPR2* genes were obtained from the leaf tissue of WS-3 rich chemo-variant by degenerate PCR and RACE methods (NCBI GenBank *Acc No. WsCPR1*: HM036710 and *WsCPR2*: GU808569). Degenerate primers based on the conserved regions were designed by employing multiple sequence alignment strategy using ClustalW. A 600 and 450 bp core fragments were obtained from the initial RT-PCR reactions. The amplicons were confirmed as segments of two isoforms of CPR by sequencing and similarity searches using BLAST. Further, extension of both amplified core fragments toward 5′ and 3′ ends by using RACE-PCR succeeded in amplification of remaining cDNA portions corresponding to two different isoforms of P450 reductase. Using GSPs designed from start codons and stop codons, 2058 bp and 2142 bp open reading frames (ORFs) were amplified from the pool of leaf cDNA designated as *WsCPR1* and *WsCPR2* respectively. Both *WsCPR1* and *WsCPR2* encoded 685 and 713 amino acid residues respectively. The first methionine as per First-AUG rule was considered as initiator codon. *WsCPR1* having upstream untranslated region (UTR; 93 bp) contained stop codons. At 3′ ends, *WsCPR1* has 357 bp non-coding region and poly-A tail while *WsCPR2* cDNA has non-coding region of 154 nucleotide residues. Similarity searches showed that both polypeptides share 80–89% identities with CPRs from other plant species. On the basis of N-terminal anchoring sequences, *WsCPR1* and *WSCPR2* conform to Ro *et al*. [18] classification of CPRs: class I and class II. *WsCPR1* has short stretch of amino acids and *WsCPR2* contained extended amino acid sequence at N-terminal. Dual CPRs are reported earlier in many plant species like *P. crispum*, *A. thaliana*, and *G. hirusitum* [19], [21], [23]. Recently, in *A. thaliana* third isoform has also been cloned and proved to be essential for embryo development [41].The well-known role of CPRs is to ensure the electron supply to P450 genes involved in metabolic pathways like biosynthesis of plant hormones and secondary metabolites such as alkaloids, sterols and brassinosteroids [42]. The existence of two divergent CPRs may likely impart *Withania* the capability to adapt their metabolic flux and survival in response to different biotic and abiotic stress.

### *In silico* Characterization of Deduced *WsCPR1* and *WsCPR2*

**Figure 2:**
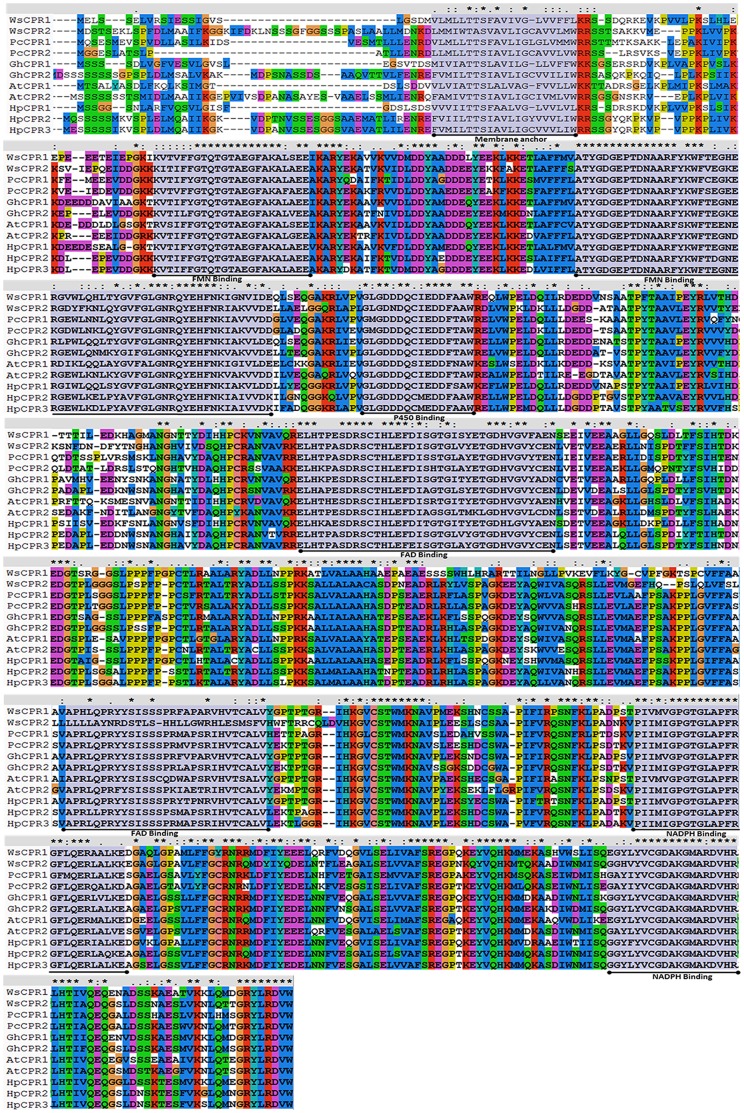
Multiple sequence alignment of deduced amino acid sequences of *WsCPR1* and *WsCPR2* with other CPR homologs using ClustalX. CPRs used for alignment were from *Withania somnifera* (*WsCPR1*: HM036710, *WsCPR2*: GU808569), *Petunia hybrida* CPR1:DQ099544, CPR2 DQ099545); *Petroselinum crispum* (CPR1:AF024635, CPR2:AF024634); *Gossipium hirsutum* (CPR1: FJ719368, CPR2:FJ719369); *Populus trichocarpa* (CPR1:XM_002307300, CPR2:XM_002329600, CPR3: AF302498); *Arabidopsis thaliana* (CPR1:X66016, CPR2: X66017). Typical conserved membrane anchor, cytochrome P450-, FMN, FAD and NADPH-binding domains characteristic for the CPR are highlighted grey in colour and underlined.

Pairwise alignment of deduced primary structures of *WsCPR1* and *WsCPR2* showed that they are 63.14% identical at nucleotide and 54.02% at amino acid level. *WsCPR1* and *WsCPR2* have predicted isoelectric points 5.38 and 5.54 and molecular masses of 76.06 and 79.05 kDa respectively. Alignment of deduced amino acid sequences of *WsCPR1* and *WsCPR2* with the known CPRs of taxonomically diverse species showed the presence of several conserved regions. At the N-terminal end of each CPR there is a membrane anchor region which is essential for normal interaction between CPR and P450 monooxygenases. Their presence was also predicted by TMHMM and SPLIT 4.0 bioinformatics programs (Figure S1). FMN domain is connected to FAD domain *via* a flexible linker region and NADPH binding domain is present near C-terminal end (Figure 2). The CPRs are supposed to have evolved from ancestral fusion of two separate genes as the FMN-containing bacterial flavodoxin (Fld) and a FAD-containing ferredoxin NADP-reductase (FNR) are found to be existing independently [43], [44]. *WsCPR1* and *WsCPR2* contain conserved acidic amino acid sequences (*WsCPR1*: LGDDDQCIEDD, *WsCPR2*: YGDGEPT) which are proposed to interact with cytochrome *c* and P450s, existing nearby FMN domain [43], [45]. The linker region which joins the FAD and FMN somehow allows the conformational changes in the position of FMN domain to interact with various P450s [46].

**Figure 3:**
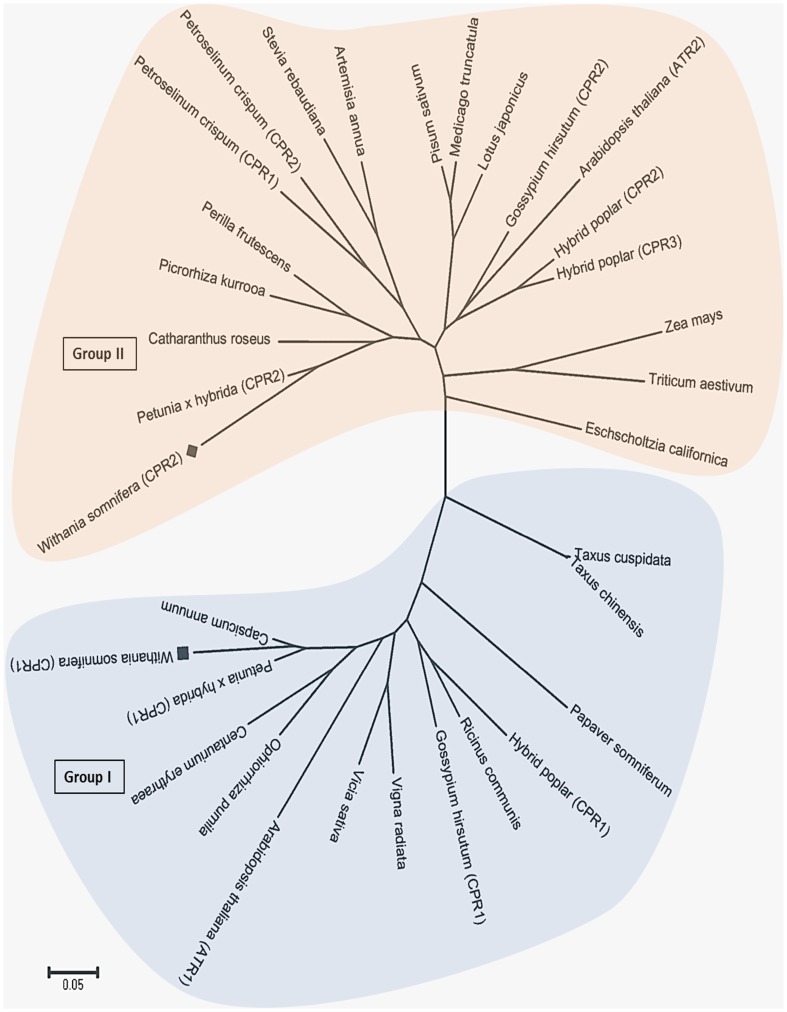
Phylogenetic analysis of deduced amino acid sequences. Phylogeny of *WsCPRs* was inferred using the Neighbor-joining method using MEGA 5 software. A total of 33 protein sequences used for analysis were from following plant species: *Withania somnifera* (WsCPR1: HM036710, WsCPR2: GU808569), *Petunia hybrida* (CPR1: DQ099544, CPR2: DQ099545); *Petroselinum crispum* (CPR1: AF024635, CPR2: AF024634); *Gossipium hirsutum* (CPR1: FJ719368, CPR2: FJ719369); *Populus trichocarpa* (CPR1: XM_002307300, CPR2: XM_002329600, CPR3: AF302498); *Arabidopsis thaliana* (CPR1: X66016, CPR2: X66017); *Capsicum annuum* (EU616557); *Vigna radiata* (P37116); *Vicia sativa* (Z26252); *Stevia rebaudiana* (DQ269454); *Ricinus communis* (XM_002514003); *Pisum sativum* (AF002698); *Picrorhiza kurroo*a (JN968968); *Artemisia annua* (EF104642); *Papaver somniferum* (U67185); *Taxus cuspidate* (AY571340); *Taxus chinensis* (AY959320); *Perilla frutescens* (GQ120439); *Ophiorrhiza pumila* (AB086169); *Medicago truncatula* (XM_003610061); *Lotus japonicas* (AB433810); *Catharanthus roseus* (Q05001); *Centaurium erythraea* (AY596976); *Zea mays* (CAC83301); *Triticum aestivum* (AGC27711) and *Eschscholzia californica* (U67186). All CPRs were grouped into two clusters where the WsCPR1 and WsCPR2 confined to their corresponding cluster like other CPRs.

**Figure 4:**
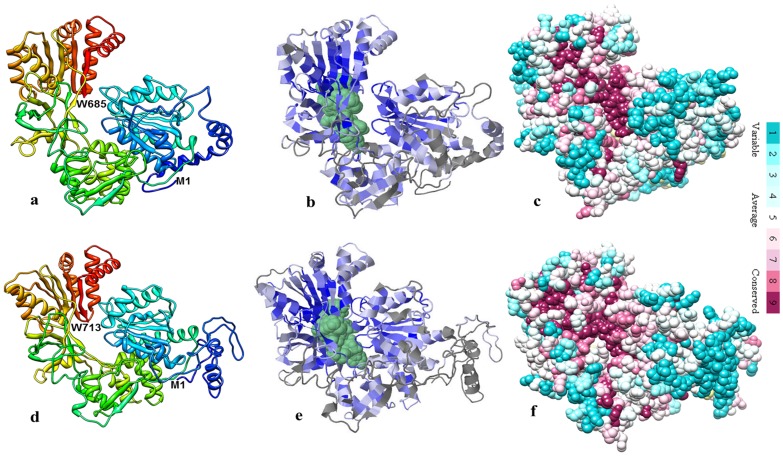
Three dimensional models and conserved residue prediction for WsCPR1 and WsCPR2. 4A & 4D: Cartoon display of the 3-D structures of WsCPR1 *and* WsCPR2 as predicted by Phyre^2^ using crystal structure of *Rattus norvagicus* (PDB ID: 1J9Z) as template. 4B & 4E: Predicted ligand (shown in green) binding sites as predicted by 3DLigandSite Web Server. 4C & 4F: Conserved residue analysis of *WsCPR1 and WsCPR2* were performed using Consurf and Conseq web servers. Residue conservation from variable to conserve is shown in blue (1) to violet (9). The residues involved in substrate binding and active site are shown in the center core of the structure.

To ascertain the degree of evolutionary relatedness, Neighbour-joining phylogenetic tree was constructed with MEGA 5 software from the ClustalW alignment of the *WsCPR1* and *WsCPR2* with number of CPR sequences of different plants retrieved from the NCBI GenBank database. The *WsCPR1* and *WsCPR2* included into separate phylogenetic groups in accordance with the amino acid similarity among their proteins (Figure 3). The CPR1 cluster contained sequences from only eudicotyledons, while the CPR2 cluster had both monocotyledons and eudicotyledon specific sequences. The presence of CPR1 and CPR2 homologs in eudicotyledons and absence of CPR1 homologs in monocotyledon strongly suggests the gene duplication event giving rise to the CPR2 cluster [43]. Secondary structure analysis of *WsCPR* proteins by SOPMA program revealed that they consist of α-helixes (43.36%; 41.23%), β-turns (4.09%; 4.49%) joined by extended strands (14.16%; 13.88%), and random coils (38.39%; 40.48%). Protein sequences when subjected to analysis for glycosylation sites were found to have two glycosylation sites for *WsCPR1* (at 278 and 514 aa residues) and one for *WsCPR2* (at 28 aa residue) respectively. For the prediction of chloroplast localization, *WsCPR* amino acid sequences were *in silico* analysed with ChloroP program. Scores for potential chloroplast targeting of CPR isoforms were, 0.463 for *WsCPR1* and *WsCPR2* scored 0.513. There was high Ser content at N-terminal in *WsCPR2*, which is predicted to be required for chloroplast targeting. Subcellular localization study of CPR proteins in Hybrid poplar has been experimentally demonstrated that they are confined to ER only [18]. However, *in silico* prediction do not rule out the possibility of their localization to chloroplast membrane also [17]. Three dimensional structural models on the basis of homology based modelling using rat CPR (PDB: IJ9Z) as template were generated by using Phyre^2^ with >90% accuracy (Figure 4A and 4D). For modelling *WsCPR1* and *WsCPR2* proteins, templates (*WsCPR1*∶1TLL, 1J9Z, 2BPO and *WsCPR2*; 2IJ2A 1TLL, 1J9Z, 2BPO) were selected based on heuristics to maximise confidence, percentage identity and alignment coverage. 63 residues for *WsCPR1* and 44 residues for *WsCPR2* were modelled by *ab initio*. In different crystallographic studies, plausible structural explanation for the mechanism of electron transfer and interaction of different domains has been elegantly described in a very precise way by some authors [47], [48], [49]. The amino acid residues involved in ligand binding were also predicted using the 3DLigandSite tool as depicted in Figure 4B and 4E. Analysis of the evolutionary conservation of *WsCPR1* and *WsCPR2* surface amino acids were performed using ConSurf program. Several residues with high scores were found to be functional and structural residues of the proteins by ConSeq servers (Figure 4C and 4F).

### Heterologous Expression in *E. coli* and Protein Purification

**Figure 5:**
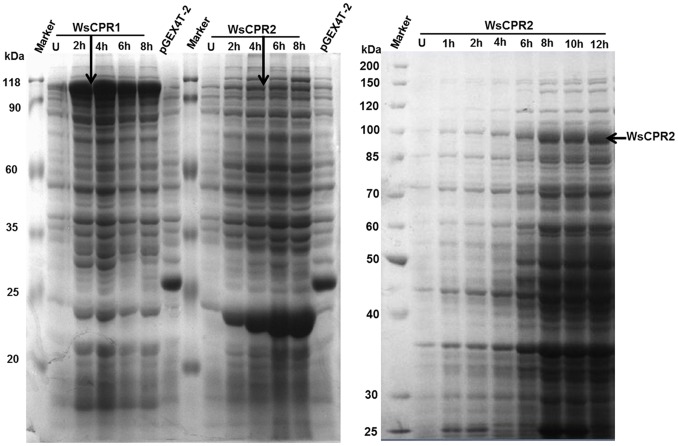
Sodium dodecyl sulphate–polyacrylamide gel electrophoresis (SDS-PAGE) of heterologously expressed WsCPR1 and WsCPR2. 5A: SDS-PAGE (10%) pattern of proteins obtained from *E.coli* BL21 (DE3) transformed with pGEX-*WsCPR1* and pGEX-*WsCPR2*. Lane 1 & 5; Standard protein markers. Lane 2 & 7; Whole cell lysate of uninduced WsCPR1 and WsCPR2, Lane 3–6 & 10–13; Cell lysate of WsCPR1 and WsCPR2 induced with 0.8 mM IPTG harvested after 2 h, 4 h, 6 h and 8 h respectively. Lane 7 & 14; Cell lysate of *E. coli* BL21 (DE3) cells containing the empty vector pGEX-4T2 obtained at 4 h post-induction with 0.8 mM IPTG. 5B: The SDS-PAGE (10%) profile of optimized WsCPR2 expression at 25°C. In different lanes the samples were harvested at different time periods as indicated above. The highest expression was observed with 0.8 mM IPTG after 12 h of induction.

**Figure 6:**
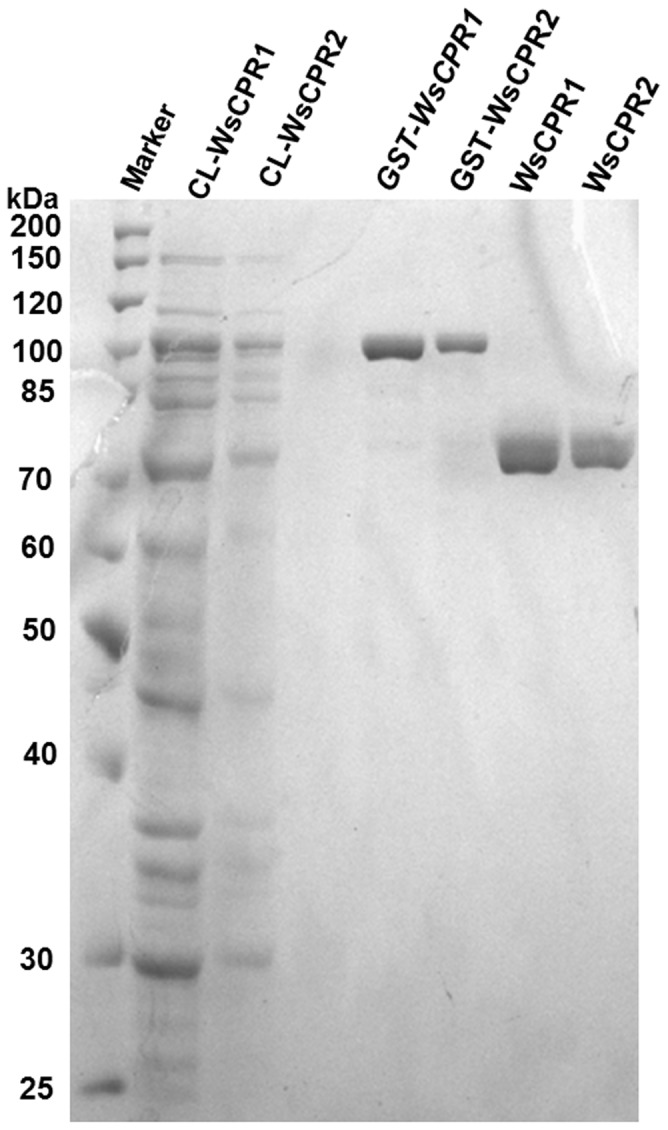
SDS-PAGE profile of purified recombinant proteins. SDS-PAGE (10%) of purified recombinant proteins from *E.coli* BL-21 transformed with pGEX-*WsCPR1* and pGEX-*WsCPR2*. Lane 1; Standard protein markers, Lane 2&3; Cell lysate (CL) of WsCPR1 and WsCPR2 expressing cells remained after incubation with GST-beads, Lane 4; Purified recombinant GST-fused WsCPR1, Lane 5; Purified recombinant GST fused WsCPR2, Lane 6; Purified WsCPR1 after removal of GST using thrombin and Lane 7; Purified CPR2 after removal of GST.

To confirm whether *WsCPR1* and *WsCPR2* encode for two different proteins, both genes were expressed in *E.coli* BL21 (DE3). An IPTG inducible *E.coli* expression vector having *Tac* promoter and N-terminal GST-tag containing a thrombin recognition site was intended for expression of both the proteins. The genes of *WsCPR1* and *WsCPR2* cleaved from the pJET-*WsCPR1* and pJET-*WsCPR2* using *BamHI/XhoI* and *BamHI/NotI* were inserted into vector pGEX4T-2. The recombinant expression vectors with the inserted *WsCPR1* and *WsCPR2* were identified by PCR and digestion with *BamHI/XhoI* and *BamHI/NotI*. The highest expression of proteins was observed at 25°C using 0.8 mM ITPG after 8–12 h of induction. The fusion protein having molecular weight of ∼105 kDa was expressed by the pGEX-*WsCPR1* and detected by SDS-PAGE analysis. The molecular weight was identical to the predicted molecular mass of the recombinant protein composed of GST (26 kDa) and *WsCPR1*. Similarly, the pGEX-*WsCPR2* produced a band of fusion protein of ∼107 kDa. Furthermore, the PGEX4T-2 vector was also transformed into *E.coli* BL21 (DE3) cells and the GST protein of 26 kDa was expressed successfully (Figure 5A). Time course study revealed that the optimum expression of *WsCPR2*, as examined from SDS-PAGE profile was obtained after 12 h of induction at 25°C (Figure 5B). Previously, functional expression of CPR in *E.coli* has been reported from *Musca domestica*, human and *G. hirusitum* [23], [50], [51]. The CPR used by Wang *el al*. [46] for X-ray crystallography was also expressed in *E.coli* in truncated form. Nevertheless, in all studies, the CPRs have been purified from microsomal fraction because these are localized to membrane. The cloning of *WsCPR1* and *WsCPR2* in pGEX4T-2 having N-terminal GST-tags allowed their expression in soluble fraction along with their membrane anchor regions. Based on the principle of affinity chromatography, recombinant fusion proteins having GST-tags were purified using glutathione sepharose beads. Initially, their expression was localized to inclusion bodies at 37°C but lowering the temperature to 25°C allowed protein expression in solubilised form. Protein purity of the two soluble proteins was enriched up to more than 90% in a single step. The purified fusion protein bands of *WsCPR1* at ∼105 kDa and WsCPR2 at ∼107 kDa in molecular mass were observed on SDS-PAGE (Figure 6). The *GST-WsCPR1* and *GST-WsCPR2* fusion was cleaved by thrombin protease and purified which showed intense band of ∼77 and ∼79 kDa (Figure 6) respectively. The amount of purified *WsCRP1* was 0.30 mg/ml while the *WsCPR2* was 0.14 mg/ml.

### Southern Blotting

**Figure 7:**
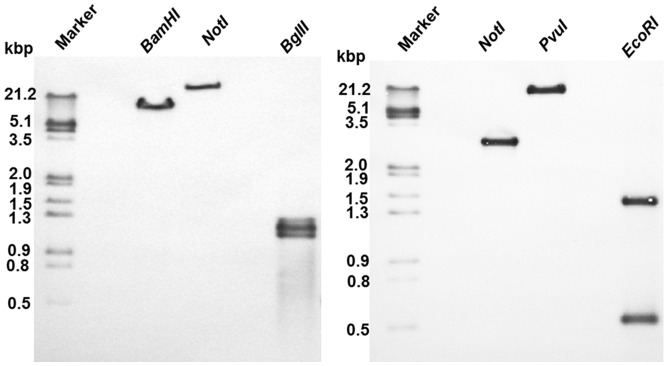
Southern blot analysis of genomic DNA. A&B: Southern blot analysis of total DNA using *WsCPR1* and *WsCPR2* as a probe. Total DNA (30 µg) isolated from *Withania somnifera* was digested with the indicated restriction enzymes, The digested samples were electrophoresed on 0.8% agarose gel, blotted onto nylon membrane and subjected to hybridisation using DIG-labelled ORF of *WsCPR1* and *WsCPR2* as probes. First lane contains molecular markers with indicated molecular weight on the left side.

To determine the gene copy number and further validation of two paralogs of CPR in *W. somnifera*, southern blot analysis was carried out under high stringency conditions using DIG-labelled full length probes for *WsCPR1* and *WsCPR2* [21]. A set of three restriction enzymes for each CPR was used in the study. Among them two enzymes have no restriction sites in coding sequence of genes and one enzyme has one cutting site for both *WsCPR1* and *WsCPR2.* The digested genomic DNA was transferred to positively charged membrane and hybridised to probes. In *WsCPR1*, one band was scored in *BamHI* and *NotI* digested DNA and two bands were detected with *BglII* digestion (Figure 7A). Similarly for *WsCPR2* the single bands were scored for DNA digested with *NotI, PvuI* enzymes and two bands for *EcoRI* (Figure 7B). This indicates that probes of *WsCPR1* and *WsCPR2* hybridised to their respective genes. The results obtained thus suggest that *Withania* genome contains two paralogs of P450 reductase and single allele for both *WsCPR1* and *WsCPR2.*

### NADPH-reductase Assays

It has been earlier demonstrated that CPR1 and CPR2 from different plant species have different specific activities and most of them have been assayed using microsomal fraction or truncated polypeptide (without membrane anchor). In this investigation, we were able to purify the proteins with membrane anchors. Purified WsCPR1 and WsCPR2 used NADPH as electron donor for reducing its substrate cytochrome *c* where WsCPR1 was observed to possess higher specific activity of 7.56±0.06 µmol min^−1^ mg^−1^ while as specific activity of WsCPR2 was 6.83±0.06 µmol min^−1^ mg^−1^ (Table 2). For kinetic studies of WsCPRs, the enzyme and NADPH were kept constant whereas the concentration of cytochrome *c* was taken in increasing order. As the substrate concentration was increased, the amount of products produced also increased. V_max_ of each purified protein was also calculated as shown in Table 2.This was explained by Michaelis-Menten plot (Figure S2). The apparent K_m_ value for WsCPR1 was 5.06±0.30 µM which showed its high affinity towards cytochrome *c* while for WsCPR2 K_m_ value was 6.48±0.33 µM. It has been shown that addition of FMN to the reaction mixture increases the specific activities of other CPRs which is mainly due to depletion of FMN domain during the isolation of microsomal fraction [23], [44]. Contrary to these observations, addition of FMN to WsCPR1 and WsCPR2 did not affect the activity as the purified proteins contained all the domains intact including membrane anchor.

**Table 2:** Specific activities and of kinetic constants of WsCPR1 and WsCPR2.

		NADPH	
	Specific activity µmol min^−1 ^mg^−1^)	V_max_ (µmol min^−1 ^mg^−1^)	K_m_ (µM)
**WsCPR1**	7.56±0.06	8.96±0.06	5.06±0.30
**WsCPR2**	6.83±0.06	10.86±0.07	6.48±0.33

For measuring specific activities, cytochrome *c* (20 µM) was reduced in presence NADPH (20 µM) as electron donor. Kinetic parameters for NADPH were studied in reaction mixture containing different concentrations of cytochrome *c* (10–250 µM). Values were obtained by non-linear regression of Michaelis-Menten plots and are presented as mean±SE.

### Tissue-specific Expression Analysis Using qPCR

**Figure 8:**
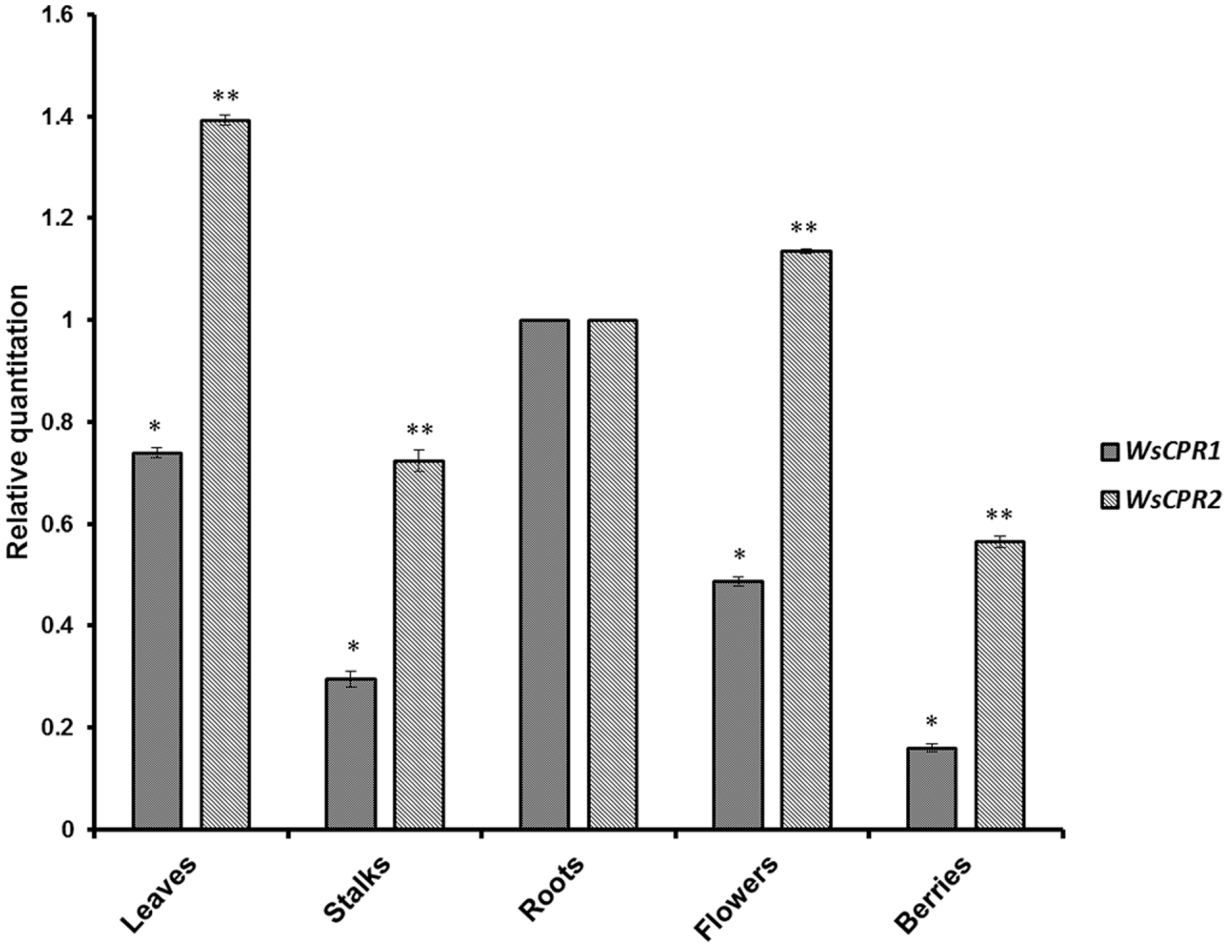
Tissue-specific real-time expression analysis. Quantitative assessment of the expression of *WsCPR1* and *WsCPR2* in different tissues of *Withania somnifera.* Data were compared and analysed with analysis of variance (*ANOVA*). Values are means, with standard errors indicated by bars, representing three independent biological samples, each with three technical replicates. Differences were scored as statistical significance at *p<0.05 and **p<0.01 levels.

To study the *WsCPR1* and *WsCPR2* gene expression pattern and levels in different tissues of *W. Somnifera*, total RNA of leaves, stalks, roots, flowers and berries (unripen) from four month old plant was used as template for quantitative real-time PCR. The results showed that *WsCPR1* and *WsCPR2* expressed constitutively with varying expression levels in different tissues as depicted in Figure 8. *WsCPR2* was found to be transcribing more in all tissues in comparison to *WsCPR1.* However, highest expression of CPR1 was observed in roots among all the tissues. The expression pattern of *WsCPR2* is in agreement with the higher content of withanolides in leaves of *W. somnifera* as reported earlier [30], [38] and probably indicates their involvement to meet the high reductive demand of different P450 monooxygenases for driving the biosynthesis of withanolides. Various steroidal lactone triterpenoids including withanolides are synthesized *via* both MVA and DOXP pathways involving CPR dependent monooxygenases like squalene epoxidase and obtusifoliol-14-demethylase [12]. Similarly, the expression analysis of obtusifoliol-14-demthylase (*CYP51*) and sterol methyl transferase (*SMT-1*) has been preponderant in leaves of *W. somnifera* than in other tissues [52]. mRNA levels of multiple orthologs of different plants like *P. crispum, A. thaliana* and Hybrid poplar varied in relation to tissues analysed depending on the species [18], [19], [21]. However, higher transcript levels of CPR2 in flowers and stems from *A. thaliana* were apparently matched by higher expression of P450s. This possibly indicates that multiple paralogs may serve as electron donors to large number of cytochrome P450s involved in the biosynthesis of variety of natural products.

### Effect of Elicitors on *WsCPRs* and Withanolides

**Figure 9:**
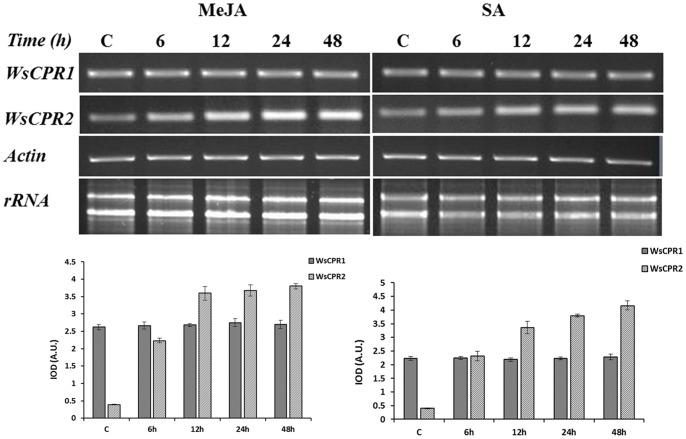
Figure **9.** **Time course effect of elicitor treatments on expression profiles of** ***WsCPRs*** **paralogs.** 9A: Time courses of *WsCPR1* and *WsCPR2* expression in micropropagated *Withania somnifera* induced by methyl jasmonate (MeJA; 0.1 mM) and salicylic acid (SA; 0.1 mM) treatments. The numbers above indicate the different time points in hours. MeJA and SA were added to the micro-propagated plantlets precultured for 2 wk on Hoagland’s medium. Total RNA from each sample was used for quantitation. Actin was kept as internal control. 9B: Expression profiles obtained by densitometric quantification of band intensities. Experiments were performed in triplicate with similar results; error bars indicate ± standard deviation of the mean. IOD, integrated optical density; A.U., arbitrary units.

**Figure 10:**
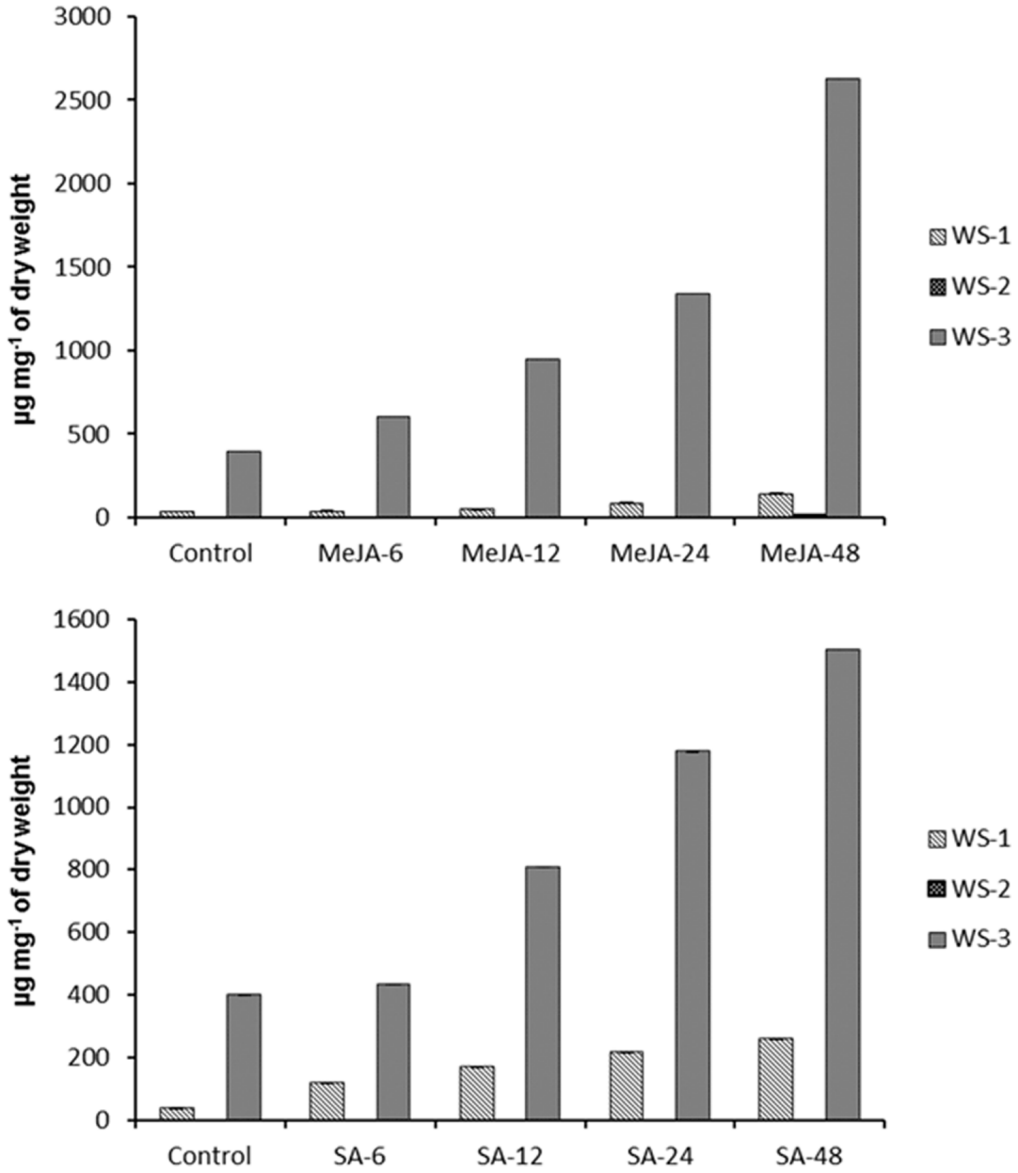
Time course effect of elicitor treatments on withanolides accumulation. 10A: Effect of methyl jasmonate (MeJA) treatment on withanolides accumulation at different time intervals. HPLC analysis demonstrated the change in three key withanolides of withanolide A (WS-1), withanone (WS-2) and withaferin A (WS-3) at 6, 12, 24 and 48 h after treatments of micro-shoots with 0.1 mM MeJA. WS-3 was observed to be enhanced more with respect to WS-1 while WS-2 was detected in sample harvested after 48 h. All values obtained were means of triplicate with standard errors. Time course accumulation of WS-1 and WS-3 was statistically significant at *p*<0.01 level. 10B: Effect of salicylic acid (SA) on withanolide accumulation at different time interval. The WS-3 level was also up-regulated in salicylic acid treated samples but WS-1 was enhanced more in comparison to methyl jasmonate (MeJA) treated samples. All values obtained were means of triplicate with standard errors. Time course accumulation of WS-1 and WS-3 was statistically significant at *p*<0.001 level.

*In vitro* cultures established *via* micro-propagation were used for MeJA and SA treatments. Micro-shoots were grown for 2 wk in Hoagland suspension medium. Subsequently, established shoot cultures were supplemented with either MJ (0.1 mM) or SA (1 mM) for 48 h. The untreated were kept as control. Samples were harvested after 6, 12, 24, and 48 h of interval. Equal amounts of DNase-treated RNA (1 µg) of control and treated samples were converted into cDNA. The effect of MJ and SA on expression profile of *WsCPR1* and *WsCPR2* was studied using semi-quantitative PCR and densitometric quantitation of relative band intensities was normalized to relative optical unit of actin (Figure 9A and 9B). The treatments with MeJA and SA resulted in induction of *WsCPR2* while the expression of *WsCPR1* remained unchanged. These observations are in agreement with earlier report on *C. erythrium* where only CPR2 was induced on treatment with MeJA [22]. In *A*. *thaliana* and *G. hirsutum*, CPR1 expresses constitutively while CPR2 was induced by environmental stimuli, such as wounding and light treatments [21]. MeJA and SA are generally considered to modulate expression of genes involved in defence responses, flowering and senescence including the genes that code for enzymes catalysing the formation of secondary metabolites [53], [54]. Treatment of these signalling molecules has been reported to enhance the biosynthesis of triterpenes in some medicinal plants like *P. ginseng* and *Centella asiatica* [55], [56]. The elicitors strongly enhanced the transcript level of *WsCPR2* which had a positive influence on withanolides accumulation. To establish a correlation between expression profiles and metabolite flux, withanolides extracted from the treated samples were subjected to HPLC analysis (Figure S3). There was increase in WS-1 and WS-3 over a period of time in response to elicitor treatments. In MeJA treated samples there was a significant increase in WS-1 (36.669±0.59−141.533±0.42 µg mg^−1^ of dry weight) and WS-3 (396.37±0.44−2629.397±0.41 µg mg^−1^ of dry weight) while as WS-2 accumulated meagrely (13.818±0.14 µg mg^−1^ of dry weight) after 48 h (Figure 10A). SA treated samples showed marked increase in both WS-1 (36.669±0.59−257.546±0.29 µg mg^−1^ of dry weight) and WS-3(396.37±0.44−1505.463±0.41 µg mg^−1^ of dry weight) with no traces of WS-2 (Figure 10B). Absence or low production of WS-2 is possibly linked to inherently lower levels of WS-2 accumulation even in cultivated accessions of *W. somnifera* [38].In one of our previous reports, MeJA and SA up-regulated the expression of squalene synthase gene which plays an important regulatory role in the phytosterol biosynthetic pathway [30]. These elicitors are important components of signal transduction cascades activating plant’s defence response against pathogen attack and often result in increased metabolite accumulation.

### Conclusion

Pharmacological studies are positioning withanolides as promising lead molecules for screening against various critical diseases and ailments. Chemically these compounds are well investigated but there exists sparse information regarding their biosynthesis. Understanding of various enzymatic or regulatory steps involved in secondary metabolite biosynthesis is a prerequisite for metabolic engineering. Pathway intensification leading to enhanced production of metabolites in host plant or heterologous production in microbial systems can prove to be important for their scalable production. Keeping this perspective in view, we have successfully cloned and characterized two paralogs of CPR. The fully functional proteins were purified from bacterial extract and enzymatically assayed using cytochrome *c* as substrate. Further, validation of CPR isoforms was done using southern blot analysis which confirmed that two separate genes exist in *Withania* genome. Expression pattern for *WsCPR1* and *WsCPR2* in different plant parts was studied using quantitative real-time PCR. Effect of MeJA and SA and their correlation with withanolides production indicates toward the possible role of CPR in metabolic regulation. The present investigation also suggests that CPRs express constitutively and at least one CPR is inducible. All the inducible CPRs reported so far belong to class II. CPRs are the most important class of enzymes and modulate the fate of various P450 involved in primary and secondary metabolite synthesis. For homologous modulation of withanolide biosynthetic pathway, we have established an efficient *Agrobacterium* transformation system (unpublished). The *Agrobacterium* mediated transformation protocol for *Withania* can be deployed to understand the regulatory role of CPRs for enhanced production of withanolides as higher transcript levels of key regulatory genes have a cascading influence on the up-regulation of downstream genes thus increasing the overall metabolite levels. We have already cloned and characterized three more withanolide biosynthetic pathway genes which include squalene synthase [30], squalene epoxidase [57], cycloartenol synthase (NCBI GenBank *Acc. No.* GU590576). Characterization of *W. somnifera* NADPH-P450 reductases may further facilitate elucidation of biosynthetic pathway of different withanolides.

## Supporting Information

Figure S1 **Protpram and THMM prediction of WsCPR1 and WsCPR2.**
(TIF)

Figure S2 **Kinetic study of WsCPR1 and WsCPR2.**
(TIF)

Figure S3 **HPLC chromatograms of elicitor treated samples.**
(TIF)
