# Supplementary figures and images for: NADPH-Cytochrome P450 Reductase: Molecular Cloning and Functional Characterization of Two Paralogs from Withania somnifera (L.) Dunal
Source: PLoS One. 2013 Feb 21;8(2):e57068. doi: 10.1371/journal.pone.0057068 (PMC3578826; doi:10.1371/journal.pone.0057068)

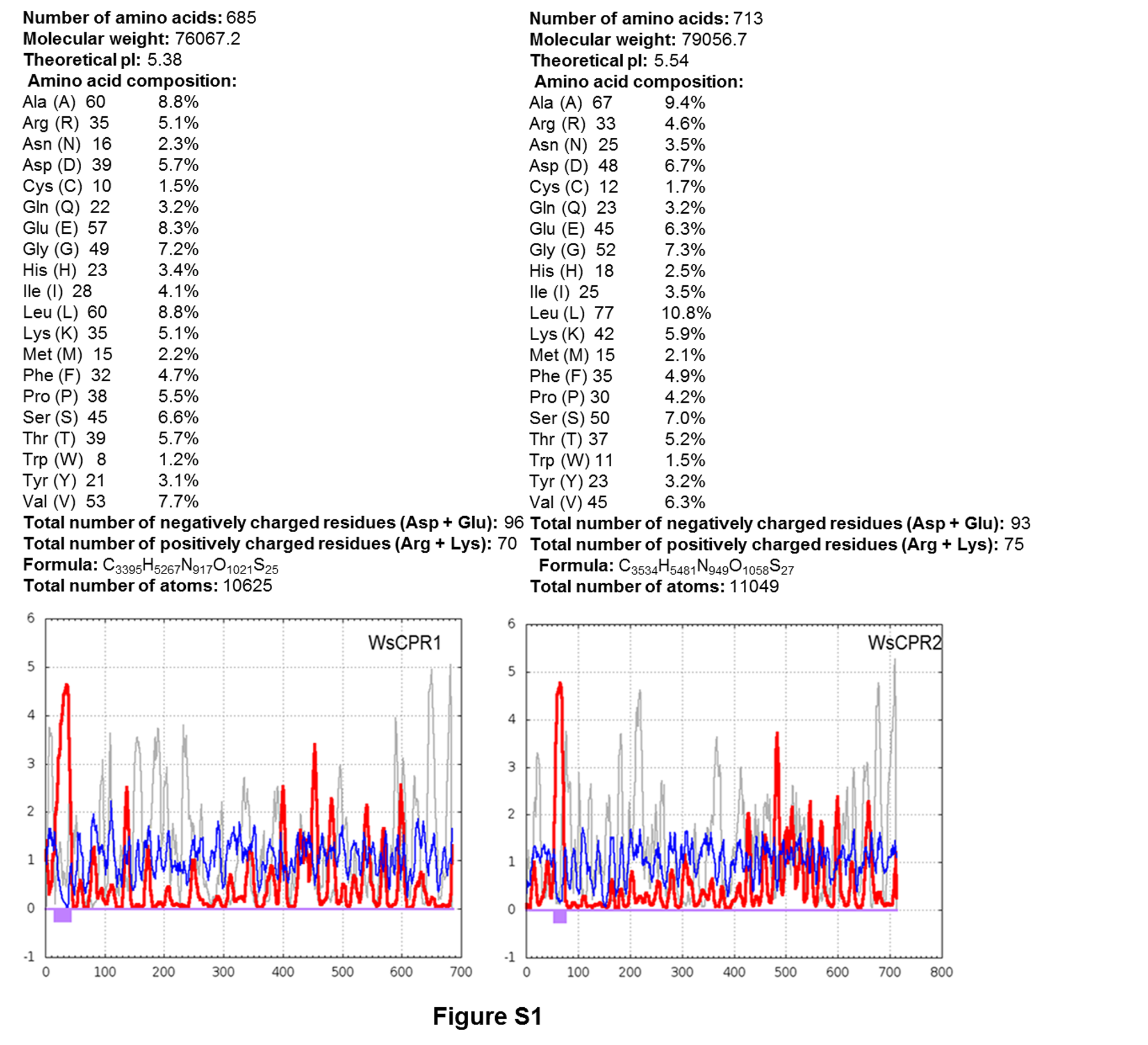

Supplement: Figure S1 — Protpram and THMM prediction of WsCPR1 and WsCPR2. (TIF) [file pone.0057068.s001.tif]

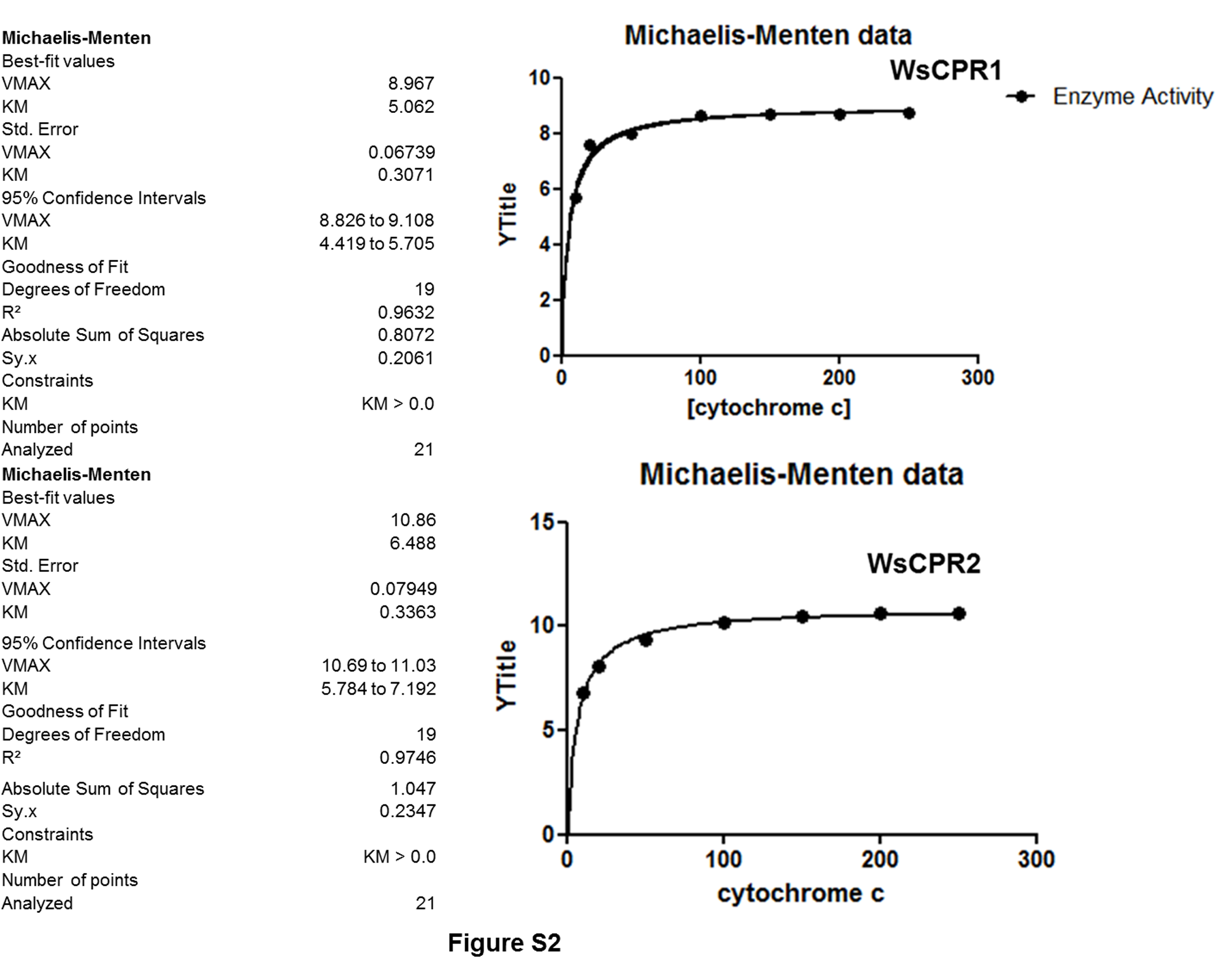

Supplement: Figure S2 — Kinetic study of WsCPR1 and WsCPR2. (TIF) [file pone.0057068.s002.tif]

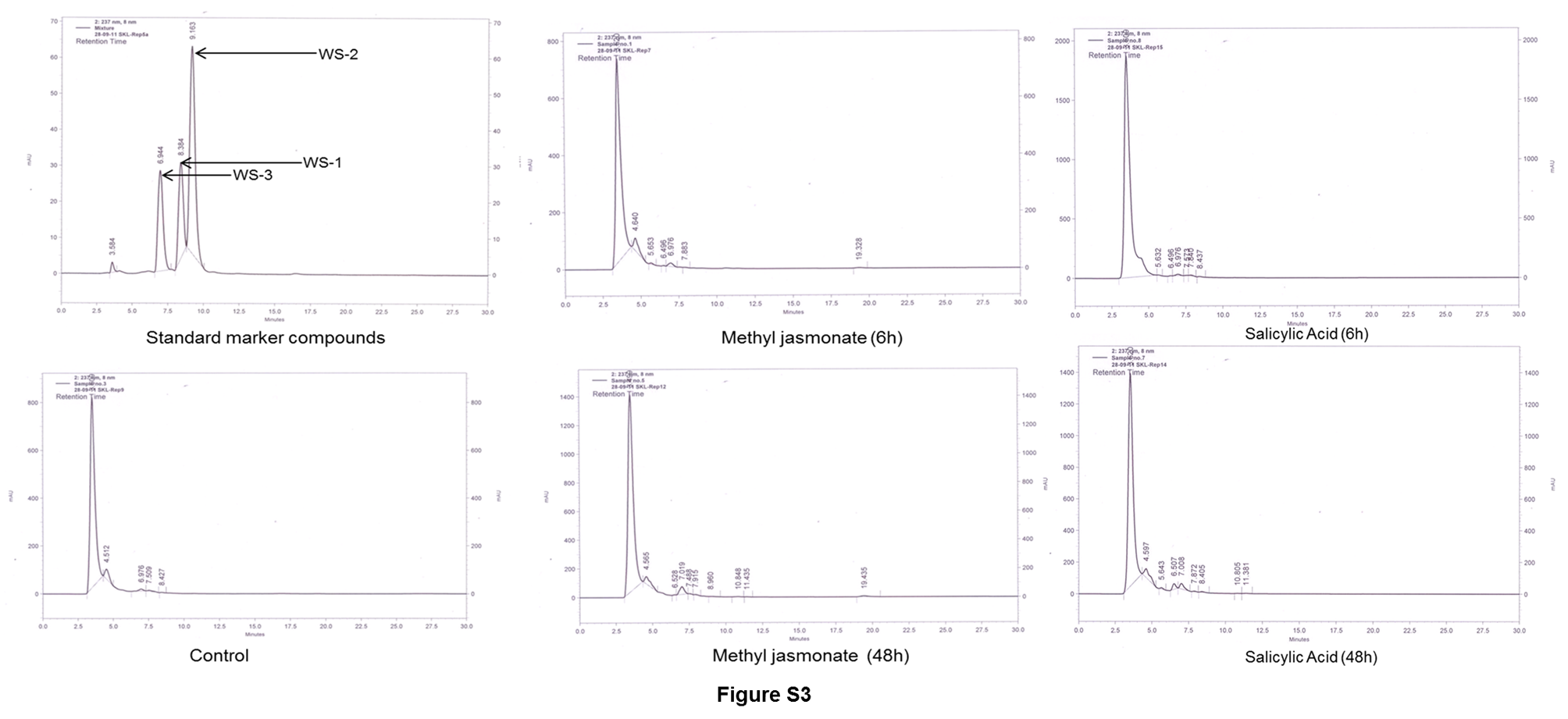

Supplement: Figure S3 — HPLC chromatograms of elicitor treated samples. (TIF) [file pone.0057068.s003.tif]
